# 

**Published:** 1921-04-30

**Authors:** 


### Future International Anti-Tuberculosis Conferences.

As our readers are aware, last autumn a conference of tuberculosis workers from some of the countries within the League of Nations was held in Paris. It was then proposed to set up an Anti-Tuberculosis Union of these countries, with headquarters at Geneva, and the next year's meeting (that is, for 1921) was fixed for London. We hear that since the Paris gathering a feeling has become manifest on the part of some British workers that all nations should meet at London, in place of the invitation being limited to those only within the League of Nations—a body, by the way, which does not include America. It may further be surmised that opposition to such an amendment of the original plan comes mostly from the French profession. What the upshot will be remains to be seen.

### Inspection of Exported Meat Products.

The Ministry of Health is strengthening its inspection of meat-food products from this country to Canada. In 1908 the late Local Government Board had the matter in hand and obtained sanction for the local medical officers of health to act as certifying officers in connection with the official certification of meat products intended for export. The war naturally put a stop to this trade, but now there is every possibility of it being renewed with the Colonies. Bearing this in mind the Ministry have taken steps to ascertain whether the medical officers, who had formerly acted in this capacity, would consent to resume their duties, and in all cases where this was required the consent has been given willingly.

### Matrons' and Sisters' Appointments.

Wellhouse Hospital, Barnet.—Miss H. M. Newton, R.R.C., has been appointed matron. She was trained at the Leeds Township Infirmary, has been assistant matron of Northumberland War Hospital, and superintendent nurse of Coventry Infirmary.

Birmingham Ear and Throat Hospital, Edmund Street.—Miss A. Strachan has been appointed matron. She was trained at the St. Helen's Hospital, Lancs. where she was later sister. She has been matron of Wallasey Cottage Hospital, theatre sister 1st Western General Hospital, matron of Warrington Infirmary, and assistant matron of Lincoln County Hospital.

Watford District Hospital.—Miss H. McKinley has been appointed matron. She was trained at Guy's Hospital, has been matron of Bexley Hospital, of Manchester Southern Hospital, assistant matron of Edmonton Military Hospital and at Middlesex Hospital, and is at present night sister at the Metropolitan Hospital. During the war she served in Belgium and France.

Brecknock County and Borough Infirmary.—Miss E. A. Shipp has been appointed matron. She was trained at Lincoln County Hospital, has been night superintendent of the West Norfolk and Lynn Hospital, King's Lynn, and matron of the Cottage Hospital, Clevedon. From 1914 to 1919 she was on active service in France and on the hospital ship *Asturias*.

Farnborough Cottage Hospital.—Miss Margaret W. Bevan has been appointed matron. She was trained at the Beckett Hospital, Barnsley, where she was later ward sister, theatre sister, and assistant matron; she has been sister at the Welsh Hospital, Netley, charge sister, Q.A.I.M.N.S.R., in India and Mesopotamia, and home sister at the Borough of Hornsey Isolation Hospital. She is a member of the College of Nursing.

Stockport Infirmary.—Miss G. M. Hooper has been appointed assistant matron. She was trained at Bolton Infirmary and Dispensary, has been staff nurse at the Hospital for Women and Children, Leeds, out-patient nurse at Clayton Hospital, Wakefield, sister at York County Hospital, home and night sister at Royal Berks Hospital, Reading, assistant matron at Royal Victoria and West Hants Hospital, Boscombe, and home sister at the General Hospital, Nottingham.

Newbury District Hospital, Berks.—Miss George, A.R.R.C., has been appointed matron. She was trained at the Great Northern Central Hospital, where she was successively sister of the women's medical ward and of the men's surgical ward, and (after service in France as theatre sister with Lady Bagot's Unit) ward sister of the military wards; she was later night superintendent and out-patient sister at the Throat Hospital, Golden Square, and assistant matron at the Military Hospital, Edmonton.

### General Nursing Council for Scotland.

At the meeting of the General Nursing Council for Scotland, held at 13 Melville Street, Edinburgh, on Wednesday, April 20, the appointment to the Council, as a nominee of the Scottish Board of Health, of Miss N. M. Fraser, matron of Gray's Hospital, Elgin, in room of Miss Janet Melrose, resigned, was announced. The Draft Rules for existing nurses were again considered and were approved, subject to certain minor alterations.

The Council considered the question of jurisdiction, and it was felt most desirable that an understanding should be come to with the General Nursing Council for England and Wales in regard to this matter. It was arranged that Miss Milnes, the Vice-Chairman of the Council, should attend the meeting in London on the following day with the representatives of the English and Irish Nursing Councils.

The position of nurses now commencing training was considered, and it was resolved that up to April 1, 1924, an applicant who had passed the first part of the examination of any existing examining body should be exempted from passing the first or intermediate examination provided by the Council's Draft Rules in regard to future nurses.

### St. Thomas's Hospital.

In connection with St. Thomas's Hospital appeal for £100,000, a letter has been received from an ex-police constable who had previously sent £10, but, on further consideration, enclosed £100 in recognition of benefits received. The police are greatly interested in St. Thomas's Hospital, and the Metropolitan Police Minstrels amused an audience of over 1,000 people who patronised their concert recently given at the Lambeth Baths in aid of the Mayor of Lambeth's fund.

THE

### COLLEGE OF NURSING

(Limited by Guarantee.)

### What the Local Centres are Doing.

### LONDON CENTRE.

(Secretary, Miss Bompas, 7 Henrietta Street, W. 1.)

Thursday, May 5, 8 p.m.—Annual General Meeting at the College of Ambulance, 56 Queen Anne Street, W. 1.

### BIRMINGHAM THREE COUNTIES CENTRE.

(Hon. Secretary, Mrs. Glegg, 84 Hagley Road, Edgbaston.)

Saturday, April 30, 3 p.m.—Annual Meeting in the Lecture Theatre of the General Hospital, Birmingham (by kind permission of the Governors). Agenda: To receive report and accounts, and elect honorary officers and Executive Committee. At the conclusion of the meeting tea will be served.

### ABERDEEN CENTRE.

(Hon. Secretary, Mrs. England, 337 Clifton Road.)

The annual meeting was held on April 21 at the Cowdray Club. A membership of seventy-six was reported. The following were elected for the year: *President*: Viscountess Cowdray; *Local Representative*: Miss Evelyn Hill (Royal Hospital for Sick Children); *Hon. Secretary and Treasurer*: Mrs. England; *Executive Committee*: Misses Leslie Scott, MacMaster, Munro (all re-elected), Laing, Forbes, Craig, R.R.C., and Mrs. Ogston; *Auditor*: Mr. Macbane. The new Superintendent-Secretary of the Club, Miss Courtier Dutton, was introduced. Great regret was expressed at Miss Edmondson's resignation of the offices of Local Representative and Hon. Secretary.

### YORKSHIRE CENTRE, LEEDS.

(Hon. Secretary, Miss Lindall, Hospital for Women and Children.)

The third annual meeting was held at Collinson's Café on April 21, Miss Innes, R.R.C., presiding. The Hon. Treasurer submitted the balance-sheet, which showed a substantial balance in hand. The Secretary reported that the membership of the Centre was approximately 260; ninety-nine new members were admitted during the year, and seventy-three members left the Centre during the year for various reasons. The use of the Outlook Club, Greek Street, Leeds, where Centre members can have lunch and tea in the restaurant at a nominal charge, has been much appreciated by Leeds school nurses. For the past twelve months there has been a daily average of six members in the Club at a time, and three members are now resident at the Hostel in Cavendish Road, Leeds. The following were elected by ballot for the ensuing year:—*President and Local Representative*: Miss Innes, R.R.C.; *Hon. Treasurer*: Miss Edwards; *Hon. Secretary*: Miss Lindall, who has filled this office since the inauguration of the Centre, desired to resign, but has consented to hold office for another year, Miss Bayly, A.R.R.C., to act as Assistant Secretary; *Executive Committee*: Miss Barry, R.R.C. (Royal Infirmary, Huddersfield), Miss Cameron (Clayton Hospital, Wakefield), Miss Harkin (Township Infirmary, Leeds), Miss Hills, R.R.C. (Royal Infirmary, Halifax), Miss McQueen (Ministry of Pensions Hospital, Beckett's Park, Leeds), Miss Smeeton, A.R.R.C. (Killingbeck Sanatorium, Leeds), Miss Sutherland (Woodlands Convalescent Hospital, Rawdon, near Leeds), Miss Whittles, A.R.R.C. (The Belmont Nursing Home, Belmont Grove, Leeds). They represent Huddersfield, Wakefield, Poor-Law, Halifax, Pensions Hospitals, Public Health, Bradford, and Private Nursing respectively.

### £50 SCHOLARSHIP.

At the annual meeting of the Leeds Centre it was resolved to offer a scholarship of £50 for the year 1921-22 towards the cost of training a sister-tutor (being a Leeds Centre member) at the University. The member must have been trained in Leeds, Bradford, Wakefield, and district, or have worked for one year in one of these towns.

### Answers to Correspondents.

Duties of Wardmaid.—The usual duties of a wardmaid include polishing, dusting, sweeping, washing-up, attending to the fires, and the keeping clean generally of her appointed ward and its offices. The duties of a nurse in a home for the aged and infirm would be the same as those required of a nurse attendant upon any patient except that they may be a little more exacting. We regret we do not give postal replies. A coupon should be enclosed with each inquiry.—J. A. E.

Post as Male Nurse.—Your experience of nursing in the Army would undoubtedly be of service to you, though apparently your training as a R.A.M.C. orderly is not complete. You might apply to the National Hospital for Paralysed and Epileptic, Queen's Square, Bloomsbury, W.C., where you would probably be accepted for the training given at this institution. You should have no difficulty at all in obtaining admission to one of the mental hospitals or asylums as a nurse-probationer. We suggest you obtain the book, "How to Become a Nurse," published by The Scientific Press, Ltd., 28-29 Southampton Street, Strand, W.C. 2, price 2s. 10d., post free. In this you will find full particulars upon all the points referred to, together with a list of institutions.—Student.

### A Directory of District Nursing.

Not the least useful of the publications of the Central Council for District Nursing in London is the issue of a Directory of District Nursing for London. This was first published in 1917, when it contained the names of 20,000 streets and places, the locality, postal district, city or borough, and Poor-law union in which each is situate, and the nurse or nursing association available for each. Corrections and additions are issued as required in the form of a supplement, so that this Directory may provide the social worker with a complete record of the district-nursing service available for each street throughout London, including the meanest as well as the more well-to-do streets, the former being no less important than the latter. The 1921 Supplement has just been published, and may be had for 4d. post free from the Central Council for District Nursing in London, Ministry of Health, Whitehall. The Directory, with the Supplement, costs 2s. 9d.

### Obituary.

We deeply regret to announce the death at Liverpool, on April 20, of Miss Emily Elizabeth Fletcher, R.R.C., Matron of Highbury, Uffculme, and Sorrento Hospitals, Ministry of Pensions, Birmingham. Miss Fletcher was Matron of the 2nd Western General Hospital, Manchester, from 1914 to 1919, and was previously assistant matron at the Royal Albert Edward Infirmary, Wigan, where she was trained. She was held in high esteem and affection by all who worked under her, and her loss will be keenly felt. She was buried in the Parish Churchyard, Winslow, Bucks, on Saturday, April 23, and at 7.30 a.m. of the same day a Memorial Service was held at Highbury, followed by a celebration of Holy Communion. Both of these services were conducted by the Venerable Archdeacon Hopton, and were largely attended by the staff and patients.

### Forthcoming Events.

Italian Hospital, Queen Square.—Annual General Meeting of Governors at the hospital, Wednesday, May 4, 1921, 3.30 p.m. His Excellency the Italian Ambassador (President of the Hospital) will take the chair.

Hospital for Sick Children, Great Ormond Street.—Annual General Meeting, Tuesday, May 3, 4 p.m., at the hospital, H.R.H. the Prince of Wales in the chair.

Jewish Maternity, District Nursing, and Sick Room Helps Society.—Annual general meeting, Wednesday, May 4, 3.15 p.m., at 56 Westbourne Terrace, W. 2 (by kind permission of Sir Isidore and Lady Spielmann), Mr. Laurie Magnus in the chair. Speakers: Miss Lena Ashwell, the Rev. Ephraim Levine, Dr. H. H. Mills, and Mrs. Model.

April 30, 1921] [Price Twopence

### The Hospital

The College of Nursing (Limited by Guarantee) 92

What the Local Centres are Doing.

RATES OF SUBSCRIPTION (post free, payable in advance).

Three months, 3/3; six months, 6/6; twelve months, 13/-. (Should the cost of printing and paper as well as increased postage, necessitate alteration of these rates, the period paid for will be reduced proportionately on unexpired subscriptions.) THE HOSPITAL "Index" to Volume LXVIII., April to September 1920, is now ready, price 1/1 per copy, post free. Address The Manager, The Hospital, "The Hospital" Building, 28/29 Southampton Street, Strand, London, W.C. 2.

**DIRECTORY OF DISTRICT NURSING,** including 20,000 Streets and places in the administrative County of London, with the name and address of the District Nursing Association or Nurse available in each Street. Issued by the Central Council for District Nursing in London. Invaluable to all Social and Health workers. Price 2/6, by post 2/9.
Apply P. S. King & Son, Orchard House, Westminster.

### PHOTOMICROGRAPHY.

**By EDMUND J. SPITTA,**
L.R.C.P.Lond., M.R.C.S.Eng., F.R.A.S.

Size 11 in. by 8½ in.

With 41 Half-tone Reproductions from Original Negatives and 63 Illustrations in the Text.

Cloth gilt, **15/-** net. **16/-** Post free.

*OF ALL BOOKSELLERS, OR OF*

**The Scientific Press Ltd.,**
28/29 SOUTHAMPTON STREET,
STRAND, LONDON, W.C. 2.

THE
### UNIFORM SYSTEM OF ACCOUNTS FOR

**Hospitals, Orphanages, Missions, Homes, Co-operations, and all Types of Institutions.**

**By Sir HENRY BURDETT, K.C.B., K.C.V.O.**

A Complete Exposition of the Uniform System of Accounts for all types of Institutions, including up-to-date additions made and approved by King Edward VII. Hospital Fund, together with specimen rulings of the various books.

"Its advantages are well known to all interested in hospital work, and institutions which have not yet adopted it will do well to do so."—*The Times.*

***NOW READY.***

Fourth Edition. Latest, and up-to-date.

**Royal 8vo, bound in cloth boards. 5/- net. By post, 5/5.**

*Obtainable of all Booksellers, or of*
**THE SCIENTIFIC PRESS, LTD., 28/29 Southampton Street, Strand, London, W.C. 2.**

